# Human Control Model Estimation in Physical Human–Machine Interaction: A Survey

**DOI:** 10.3390/s22051732

**Published:** 2022-02-23

**Authors:** Adriano Scibilia, Nicola Pedrocchi, Luigi Fortuna

**Affiliations:** 1 Department of Electrical Electronic and Computer Engineering, University of Catania, 95125 Catania, Italy; luigi.fortuna@unict.it; 2 Institute of Intelligent Industrial Technologies and Systems for Advanced Manufacturing, National Research Council of Italy, 20133 Milano, Italy; nicola.pedrocchi@stiima.cnr.it

**Keywords:** human-in-the-loop, control modelling, man–machine interaction

## Abstract

The study of human–machine interaction as a unique control system was one of the first research interests in the engineering field, with almost a century having passed since the first works appeared in this area. At the same time, it is a crucial aspect of the most recent technological developments made in application fields such as collaborative robotics and artificial intelligence. Learning the processes and dynamics underlying human control strategies when interacting with controlled elements or objects of a different nature has been the subject of research in neuroscience, aerospace, robotics, and artificial intelligence. The cross-domain nature of this field of study can cause difficulties in finding a guiding line that links motor control theory, modelling approaches in physiological control systems, and identifying human–machine general control models in manipulative tasks. The discussed models have varying levels of complexity, from the first quasi-linear model in the frequency domain to the successive optimal control model. These models include detailed descriptions of physiologic subsystems and biomechanics. The motivation behind this work is to provide a complete view of the linear models that could be easily handled both in the time domain and in the frequency domain by using a well-established methodology in the classical linear systems and control theory.

## 1. Introduction

Modelling the human control action when interacting with a controlled machine has become an almost independent research field over the past few years, involving multiple disciplines and approaches. Neurophysiologists and cognitive scientists have improved our understanding of human perception, information processing, and control strategies with respect to prior approaches, mainly focusing on qualitative descriptions of possible human decisions and actions. Since the instrumentation and measurement techniques used in this area have dramatically improved, along with the power of computational calculations, scientists have developed functional maps of neurons and identified deep brain functions [1,2,3]. Still, the human brain’s dexterity and plasticity have many mysteries, and the question of how humans can interact by adapting themselves to unknown external dynamics remains an open issue.

Therefore, researchers have put intense effort into investigating motor control internal dynamics, perceptions, and learning. With particular reference to the study of the input–output characteristics of the motor apparatus, the concept of the internal model has allowed significant advances to be made in describing human adaptation to external dynamics and trajectory planning [4]. The concept underlying the internal model hypothesis originated in robot control. A robot needs to have knowledge about its internal kinematic model in order to perform any position, velocity, or force control action. However, this same concept was extended to human physiology when Ito [5] proposed that internal models of the limbs and connected brain regions are present in the cerebellum. The acquisition of the inverse dynamics of motor efferent systems and inverse dynamics of controlled objects has helped us to explain how it is possible to perform fast and complex movements even if time delays and low gains characterize biological feedback structures [6]. An opposite approach to the same problem relies on equilibrium-point control models [7]. The central nervous system can control muscle dynamics by simply acting on its threshold level. Predictive simulations of human movement have also been used to dissociate the contributions of neural and musculoskeletal impairments to gait deficits in cerebral palsy [8], evidencing the importance of having a precise model of involved body parts and physiological districts in order to improve the correspondence between simulated and measured data [9]. The same was found in the rehabilitation robotics field, where the mechanical impedance control parameters of a human’s upper limbs were identified to adapt the robot’s training strategy accordingly [10,11].

Modelling approaches of this type have linked mathematical descriptions typical of classical control theory with functional descriptions of the physiological systems acting during the control process, linking neurosciences with more practical engineering fields such as robotics and aerospace. Aerospace researchers were involved in the first application scenario, which motivated their interest in this topic throughout the first few decades of the last century. Starting from World War II, engineers’ and psychologists’ efforts were directed towards modelling human behavior as an inanimate feedback controller to improve the performances of pilots and bombardiers [12]. The first approach was to consider the human controller as an inanimate servomechanism represented in a simple feedback structure as a block with well-defined input(s) and output(s). In a manual human-in-the-loop control problem, the human input is an error signal, usually visual. Meanwhile, the output is human’s control actions, which provide a command to the controlled element (i.e., a gun, an aircraft, or a vehicle). The same consideration is still valid in physical human–robot interaction, especially in applications involving force or impedance control strategies. Here, continuous and compliant physical contact is required to perform cooperative tasks, which can be reduced to mutual movement between humans and robots. In this case, the human operator should deviate from the robot’s initial trajectory and impose a different one.

In such a context, the human operator will act as a proper motor controller by internally deciding the goal trajectory to perform and imposing an external force to achieve such an objective by manipulating the robot. From this point of view, the performance of the human operator can be well approximated as the action of an inanimate controller. This situation results in a simple compensatory manual control system. In [13], Hess explains this concept by giving the example of a human soldier performing a tracking task, attempting to keep a moving target within the gun’s field of view. In this case, the angular error between the target and the gun’s view fielder’s azimuth can be considered the input, while the output control action is a force acting on a simple gear mechanism. Since the soldier is modeled as an inanimate servomechanism, the mathematical representation used to describe him should be the same as a linear servomechanism: a set of linear differential equations with constant coefficients, or equivalently, a transfer function in the frequency domain. The most famous example of this kind of approach is McRuer’s crossover model, in which the human is represented as a general quasi-linear describing function. From this first approach, the development of mathematical descriptions of human controllers in a control theory fashion evolved along with new control techniques. For instance, the development of linear quadratic Gaussian control systems (LQR) led to the “optimal control model” being applied to human operator modeling. The same concept is valid for recent modelling techniques such as fuzzy control models or models based on neural networks, which followed the spread of these techniques in systems design.

However, human modelling efforts, which started from this applicative scenario, have proved to be useful in many other domains of engineering. Classic examples are display and control equipment interface design based on man–machine environment system engineering in various types of plants [14,15], and more recently, service robotics applied to healthcare [16,17]. The understanding and prevision of human action have been extensively investigated in the domain of human–robot interaction (HRI) in the last few years, being considered input information that enables the compliant and adaptive behavior of the robot. In [18], a task-adaptation framework was developed to improve robot compliance with respect to human movements. Similarly, in [19] gesture-based HRI, which would allow automatic task manager parametrization where the human could help or correct robot choices in collaborative assembly applications, was discussed. Meanwhile, refs. [20,21] tried to analyze the psychological and emotional implications of continuous interaction with a robot in a production setup. Behavioral criteria were also considered in HRI in commercial vehicles [22], where a scheme of mental state variables was used to modulate driving velocity and breaking in different moments of the day and night. Similarly, machine learning techniques were also applied to HRI intelligent transport systems [23].

Since most of these models result from very application-specific efforts, their variability causes some difficulties when trying to find common features and divide them into general categories. In other words, every effort put into the definition of human control models originated from the need to describe their behavior in a particular situation, starting from different perspectives and with different levels of abstraction. According to Rasmussen [24], human behavioral models for interacting with an aircraft can be grouped into three types: skill-based, rule-based, and knowledge-based. The human–machine system is continuously controlled in skill-based models following a mission statement. Rule-based models provide a discrete decision-making description of human behavior that is guided by a stored rule. The last category groups all the control strategies deriving from unexpected events or unfamiliar environments, in which the human operator has to avoid dangers and risks. An example can be found once again in the pilot’s control of an aircraft. Traditional control models are mainly part of the first category, while modern artificial intelligence techniques have increased the use of models based on the other two. Xu et al. [25] proposed a different classification, identifying human models based on control theory, human physiology, and intelligence techniques. Classical feedback control models such as McRuer’s crossover and optimal control models fall into the first category.

Examples of successful modelling techniques which provide a better description of all the underlying processes determining the overall human control strategies are the Hess structural model and Hosman’s descriptive model. Hess proposed a detailed description of human perception processes and inner loop feedback. At the same time, Hosman modeled the interaction between visual and vestibular inputs and their influence on the overall control strategy used. Additional models that are associated with this category are biodynamic models, which try to include the biomechanical effects of the body moving into an accelerating environment such as an aircraft or a vehicle. Equivalent to the previously described knowledge-based models, the models based on intelligent techniques include approaches dealing with uncertainty, such as fuzzy logic and neural networks. This work will focus on models based on control theory, including the physiological structures involved. These are part of the categories indicated in the first two rows of Table 1, which indicates the similarities between the two different indicated classification methods. Moreover, humans have both linear and non-linear behavior when interacting with a machine. This aspect is reflected in the classifications of control models, where both linear and non-linear dynamics are described.

In this work, our focus will be mostly be on linear models. The paper will be structured as follows: The main motor control theories will be addressed in the second section. Then, the third and fourth sections will detail the modelling approaches used for neuromuscular dynamics and human sensory systems, respectively. The fifth and last sections will describe the most important human–machine interaction models, with the dynamics described in the previous sections being represented within a general control structure.

## 2. Motor Control in the Central Nervous System

Neurophysiology researchers have widely studied motor control dynamics in the human nervous system in recent years with different approaches. One of the most promising assumes the existence of internal models of sensory motor output dynamics in the central nervous system (CNS). In [26], it is suggested that the cerebellum forms two different types of internal models. One of them is a *forward* predictive model of the motor apparatus (e.g., limbs and muscles), providing a rapid prediction of the sensory consequences of each movement. The second is a model of the time delays in the control loop (due to receptor and effector delays, axons, conductances, and cognitive processing delays). This second model delays a copy of the rapid prediction in the temporal register with actual sensory feedback from the movement. Both models can coexist and form two Smith predictors. In [27], it was experimentally verified that human subjects could estimate their hand position without visual feedback and with applied external disturbances, supporting the evidence that the central nervous system internally simulates the dynamic behavior of the motor system in planning, control, and learning. Such internal models of motor dynamics and temporal delays in the central nervous systems have been discussed extensively in the fields of cognitive science and neurophysiology. The necessity of the use of internal models in motor control has been one of the central issues of debate concerning other approaches to motor control theory, such as equilibrium-point control.

In equilibrium-point control, or threshold control theory (TCT), motor actions are controlled by changing the neuro-mechanical parameters which establish the steady state (equilibrium point), which is controlled at lower levels (e.g., muscles and limbs) by descendent systems. The neural control variables which determine the equilibrium point are identified in the *λ* model [28]. A variation in the muscle length triggers muscle activation by efferent neurons and motor units. When a muscle is quasi-statically stretched, the potential in the motoneuron membrane increases; after that, a specific threshold value is reached, and the motoneurons start to be recruited. Physiological data indicate that such a threshold length value comprises various factors aside from its central component. If the central component is *λ*, the composite value is:

(1) $$\lambda_{c}=\;\lambda -\mu \omega -\rho +\varepsilon \left(t\right)\;$$

where μ is a temporal parameter related to the dynamic sensitivity of muscle spindle afferents, ω is the velocity of the change in the muscle length, ρ is the shift in the threshold resulting from reflex inputs (such as those responsible for the inter-muscular interaction and cutaneous stimuli), and ε(t) represents temporal changes in the threshold resulting from the intrinsic properties of motoneurons. In Equation (1), both λ and μ parameters are controllable by the CNS. Therefore, according to the TCT theory, high control levels can control muscular activation, minimizing the difference between the actual length and the threshold established.

**Figure 1:**
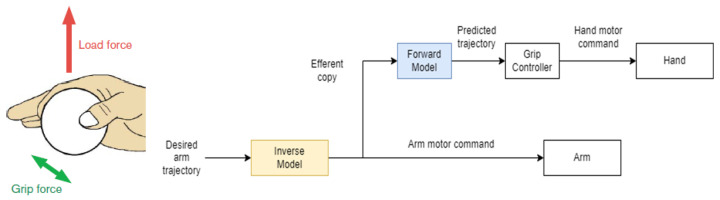
Example of how a control structure based on internal models in the cerebellum can be used to explain a human′s coordination of load and grip force in a simple grasping task.

Let us consider a situation in which the human subject is asked to hold an object; according to TCT theory, the gripping force is set in such a way that the difference between the threshold length (established by the physical shape of the object) and the actual length, which is set to be virtually inside the object, is the minimum. The result of this operation is that the object is held using the minimum quantity of gripping force. The same situation was considered from another point of view by Kawato in [29] and is shown in Figure 1. Here, the coordination of reaching and grasping, which allows using the minimum grasping force to prevent slip when lifting an object, was considered the proof of the existence of internal inverse and forward models of the limbs. When the arm grasps an object, the inverse model of the combined dynamics of the arm, hand, and object computes the necessary motor commands from the desired arm trajectory. Such commands are sent both to the arm muscles and to the dynamic forward model, which can predict the future trajectory and establish the grip force necessary to lift it, considering its friction and a safety factor.

Alternative explanations of how such predictive capabilities can be possible without internal models also exist. These are linked to the “strong predictive” and anticipatory properties manifested by biological systems. In [30], strong predictive systems are defined as those in which predictive properties are inherent in the systems′ natural dynamics and thus do not rely on internal models. At the same time, weak predictive systems are based on their internal models.

Another motor control case study that was analyzed to highlight the differences between the two approaches is the formation of an arm trajectory [31]. It is known that muscle and peripheral reflex control loops have spring-like characteristics that can pull back the limb’s joints to their equilibrium positions by generating a force directed against the sensed external perturbations. This viscoelasticity can be considered the static gain of the peripheral feedback control loop. It can be adjusted by adequately setting the associated muscle co-contraction level and the reflex gain. The equilibrium-point control hypothesis implies that through this viscoelasticity, the brain can control the movements of the limbs by simply setting a series of stable equilibrium positions aligned along the desired trajectory [32,33]. An experimental study of this concept was presented in [34], where the data of [35] were reinterpreted in an equilibrium control fashion with a straight equilibrium trajectory. However, this approach requires viscoelastic forces to increase proportionally to the movement speed, since the dynamic forces exerted to multi-joint links depend on the square of the velocity. In contrast, the alternative explanation implies that the internal model control allows accurate and fast movements, even when considering low viscoelastic forces [36,37,38]. Experimental evidence of a relatively low stiffness observed during movements performed by a well-trained subject supports the latter hypothesis [39,40]. Another step forward was to integrate the two approaches, muscle viscoelasticity and internal models, through computational models in order to efficiently learn the behavior and applications of internal models [41,42]. For example, in [39], the authors showed that intrinsic muscle stiffness is not strong enough to stabilize upright posture; thus, the usage of internal models was suggested as an alternative explanation.

## 3. Neuromuscular Dynamics Model

**Figure 2:**
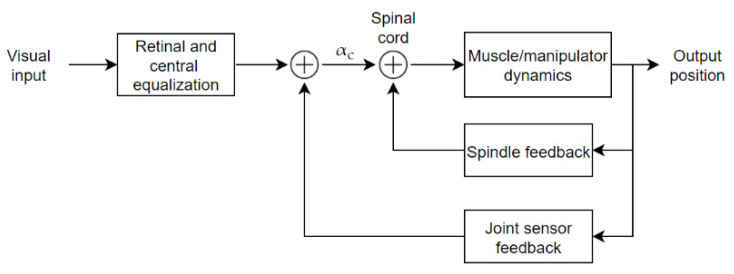
Model of subsystems contributing to neuromuscular dynamics in manipulative control tasks, as studied by McRuer et al.

The neuromuscular system dynamics model has been widely investigated in manipulation tasks starting from the early 1960s. Typically, muscles and manipulators are considered to be unique functions. An example of what can be involved in muscle-manipulator dynamics was studied in [43], and a simple block scheme of neuromuscular subsystems is shown in Figure 2. Retinal and central equalization transfer function change according to the considered forcing function forcing function dynamics, as represented by simple gain and delay factors in [43] for a rate stimulus, but change for other controlled elements. The alpha motor neuron command α_c_ is the command input from higher centers to the spinal cord. The change in the average firing rate of the alpha motor neurons involved is proportional to the driving force. The commanded force signal then goes to the muscle/manipulator block, whose dynamics, as previously mentioned, are represented by a unique transfer function that consists of a third-order system with one real root and a quadratic pair plus a time delay:

(2) $$H_{MM}=\frac{-K_{I}e^{-\tau_{\alpha }s}}{\left(1+T_{N}s\right)\left(1+\frac{2\zeta_{\alpha }}{\omega_{\alpha }}s+\frac{s^{2}}{\omega_{\alpha }^{2}}\right)}$$

where $K_{I}$ and $T_{N}$ represent, respectively, a gain factor and lag constant, while $\tau_{\alpha }$ is the motoneuron delay. The muscle characteristics are functions of the steady-state isometric tension of the muscle system operating point. The changes in this average tension are caused primarily by the discharge of the gamma motor neuron system. The effects of the gamma neuron bias signal, while not shown explicitly in Figure 1, are used to set up the spindle feedback operating point equalization, whose block also approximates the Golgi tendon force feedback. The corresponding describing function is:

(3) $$H_{SP}=\frac{K_{sp}\left(s+Z_{sp}\right)e^{-\tau_{sp}s}}{\left(s+P_{sp}\right)}$$

Here, $K_{sp}$ and $\tau_{sp}$ represent spindle gain and delay factor. The effective joint sensor provides a second feedback loop, represented by a gain factor and a time delay $K_{J}e^{-\tau_{J}s}$, operating in the frequency region of interest. Therefore, the closed-loop neuromuscular system has third-order dynamics plus a zero due to the spindle pole in the feedback loop. Data obtained by McRuer et al. [44] indicate that the muscle/manipulator dynamics for rudder pedals and hand manipulators are similar in form and numerically, despite the difference in limb size and function. In [45], Van Paassen et al. suggested an extension to the model in which the manipulator and the human arm are no longer unique blocks, but where their interaction is considered. Such analysis is helpful in application scenarios in which the human subject is operating a task while subject to acceleration (i.e., in a moving vehicle) or it is using active manipulators in which an active servo element is used to provide feedback from the controlled system or other sources in the environment. 

Research efforts have also been addressed towards the analysis of the relation of human performance in manipulative tasks with muscle fatigue dynamics. An example of such muscle fatigue and recovery models is proposed in [46], which links the maximum voluntary contraction (MCV) to the output isometric force in a cycling application. Liang et al. [47] proposed a model in which the muscle capacity after a certain number of contractions is evaluated and put in relation to an external load force. A further extension of this analysis also considered the relation of MCV with the brain effort, distinguishing between fatigued and non-fatigued motor units [48].

Other research activities rely on different types of modelling techniques, such as in [49,50], where a bond graph mathematical model was used to describe the biomechanical characteristics of the upper limbs tendons during grasping. In [51], bond graphs were used to describe the extensor mechanism of a finger, being represented as deformable strings, and assumed to pass through hooks fixed at predetermined points on rigid phalanges.

However, all the literature’s descriptive models of neuromuscular dynamics operate at high frequencies. This consideration leads to the fact that when neuromuscular dynamics are considered as an element of a more general control model, such as the ones analyzed in the next section, often only their low-frequency effects are taken into account. Such effects can be simplified as a delay element.

## 4. Sensory Dynamics

Although all sensory organs are well known individually, their dynamic behavior and joint role with the CNS in perception has been of interest in further investigations recently [52]. One example that has motivated recent interest in sensory dynamics modelling is operator disorientation. Although it is commonly taken for granted that reality can be accurately perceived, situations in which a human is subject to continuous rotations may lead to spatial disorientation. Spatial disorientation is defined as a situation in which the human operator fails to perceive their correct position, motion, or attitude. Research works in this field date back to the latest decades of the 19th century and were initiated by Ernst Mach and his colleagues through a study on vestibular and acoustic perception. However, actual progress was only obtained after almost a century, in the 1990s, when the mathematical modelling of spatial disorientation was proposed [53].

### 4.1. Visual System

The human visual system is the primary source of information in our sensory system. It has been widely studied as a mathematical model of computer vision techniques or with regard to the development of simulators for vehicles or aircraft. The importance of this sensory modality in the latter application scenario is confirmed by the fact that such a simulator very often relies on a fixed-base structure and thus does not stimulate the vestibular system. Human vision can operate mainly in two modalities: ambient or focal mode. 

The ambient mode mainly intervenes in humans’ spatial orientation capabilities. It relies on several central and peripheral vision systems inputs, such as motion, perspective, texture, and brightness gradients. The most interesting characteristic of this visual model is its capability to subconsciously process any disturbance over the input signals and provide a stable perception covering a sizeable spatial range. This information is used to determine spatial orientation by sending low-frequency, robust signals to the CNS. In contrast, other sensory systems provide high-frequency transient signals to help stabilize the perceived surrounding environment immediately after motion. The ambient mode is beneficial for perceiving distance and the angle between the operator’s plane and the ground (i.e., slant). Lone and Cooke described the possible sources contributing to the spatial disorientation (SD) of a pilot guiding a vehicle [52]. The visual perception of both slant and splay angles—respectively, the relative orientation in the vertical and horizontal plane—can lead to misjudgments in humans’ estimation of the controlled element’s actual position.

**Figure 3:**
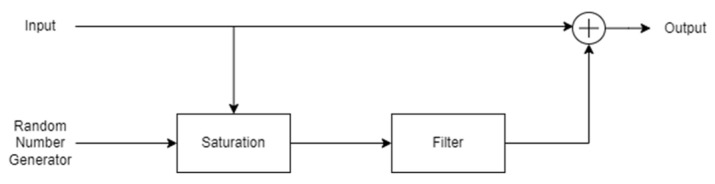
Visual cue perception model proposed by Hess.

The focal mode is linked to object identification and relies mainly on binocular signals coming from the central visual field. It also provides very detailed information at high spatial frequencies and is usually represented in conscious states [54]. Figure 3 represents the visual perception model proposed by Hess in [55], providing a simple way to model visual observation. Saturation limits can be set by considering two times the value of the random number generator input variance. Consequently, the variance determines the visual signal quality and is related to the relationship between a usable cue environment (UCE) and visual cue rating (VCR) [56,57]. Its value can be selected between the following ranges:

(4) $$\begin{array}{c} 0<\sigma_{VIS}^{2}<0.1\;\;\;\;\;\;\mathrm{if}\;\mathrm{UCE}=1\\ 0.1<\sigma_{VIS}^{2}<0.2\;\;\;\mathrm{if}\;\mathrm{UCE}=2\\ 0.2<\sigma_{VIS}^{2}<0.3\;\;\;\mathrm{if}\;\mathrm{UCE}=3 \end{array}$$

Such a parameter has been extended to task-dependent variance related to vision with multiple axes:

(5) $$\sigma_{task}^{2}=\{\begin{array}{c} \;0.01n\;\mathrm{if}\;n>1\;\\ 0\;\mathrm{if}\;n=0 \end{array}$$

where *n* is the number of controlled axes. The two terms can be incorporated into the following factor:

(6) $$f=1+10\left(\sigma_{VIS}^{2}+\sigma_{task}^{2}\right)$$

Along with the global view of vision modalities, human vision has also been studied in relation to object tracking, particularly in the computer vision and image processing fields. Nguyen et al. [58] recently developed a tracking model that utilizes spatial-temporal context information to increase tracking accuracy. Further improvements in visual tracking research have been gained by the widespread adoption of discriminative learning methods [59]. These classifiers are tasked with distinguishing between a target and its surrounding environment and are often used to ensure target tracking in the presence of occlusions [60]. The latest trend in the field is multiple object tracking [61]. The challenge, in this case, is in locating multiple objects, maintaining their identities, and yielding their trajectories given an input video (in the case of computer vision applications). Objects to label, in this case, can be pedestrians [62] or vehicles [63] in the road safety management field.

### 4.2. Vestibular System

The vestibular system is responsible for human equilibrium, postural control, and the proprioceptive sense of body motion. Anatomically, it is housed in the inner ear and divided into semicircular and otoliths canals. The otoliths perceive a sense of tilt and force, while semicircular canals help provide the sense of angular acceleration. The accurate analysis and estimation of their dynamic response have been crucial for human perception modelling when interacting with any mobile-controlled machine.

Angular motion characterized by low amplitude is limited by inherent thresholds, which are a function of the magnitude of the stimulus and its duration. Angular accelerations with a duration inferior to 10 seconds are described by Mulder’s law, with the product of angular acceleration and its duration being approximately equal to 2.5 deg/s. Therefore, a weaker acceleration requires more time to be perceived from vestibular canals. In the aerospace domain, experimental studies on human sensory thresholds for angular velocities and accelerations characterized by a prolongated duration have been carried out [64]. It is suggested that such thresholds can vary depending on the nature of the controlled elements. For example, a flight can be slightly higher with respect to a car due to more stress and, consequently, the pilot’s attention level and allocation. The pilot’s experience and training contrast this effect. In this case, the human has an accurate internal model of the machine’s dynamics, allowing them to have a certain degree of knowledge in advance and to lower this threshold.

To summarize, the workload, stress level, and training level substantially impact humans’ abilities to sense a rotational motion. It is difficult to obtain an accurate model of dynamic threshold variation because of the parameters being hard to quantify. The primary role of otoliths relates to the sense of linear accelerations and vertical motions, with threshold levels of, respectively, 0.1 g and 2 deg. Otoliths canals cannot differentiate between acceleration caused by gravity and other linear accelerations. The sensed motion should always be considered an apparent vertical motion, since there is no difference in how humans perceive tilt and linear accelerations.

Further attempts to model the vestibular system led to Hosman’s descriptive model, whose primary purpose was to integrate visual and vestibular dynamics, which will be discussed in Section 5.

### 4.3. Proprioceptive Systems

Some of the first senses to develop in a human being are tactile and proprioceptive, since they are mandatory for determining the gravity vector and consequently developing the necessary anti-gravity group of muscles, which allow us to walk. 

Proprioception, also called kinesthesis, refers to such a sensory modality, which uses muscles spindles to determine the position of the body and limbs of the subject, as well as their movements and the joint torques required to start a motion or maintain a steady position against resistive loads. In human–machine interaction, the role of tactile and proprioceptive systems is mainly linked to the force and pressure feedback that the operator has due to the physical contact with an aircraft inceptor, a vehicle steering wheel, or a robot’s end-effector. 

Pressure receptors are located within the skin all over the human body. They are of primary interest in the development of modern haptic feedback devices. For example, they can provide information about a surface texture belonging to an unknown external environment in teleoperation frameworks. 

The modelling of this system is complex because of the significant number of physical stimuli which trigger its response. Classical factors that trigger the output of the proprioceptive system directed to the CNS are relative linear and angular velocity, muscle tension, and its orientation with respect to the gravity vector. Usually, all these inputs are sensed and elaborated simultaneously. Since, as is noticeable, some of these factors also stimulate other sensory systems, the CNS combines the multiple pieces of sensory information received to develop its proprioceptive sense in case of conflict. For this reason, proprioception cannot be seen as a unique sensorial system like the visual and vestibular ones. However, it should be considered more as a sense developed as a combination of different sources of information. Hess provided a transfer-function representation of proprioceptive dynamics in his “structural model”, which will be discussed in Section 5.

### 4.4. Inter-Sensory Models

A relatively recent field of study is human cognition associated with multi-sensory stimuli. The first steps in this context were provided, once again, by neuroscientists such as Halligan [65] and Lotto [66], even if the interactions considered between senses were limited, focused on forms of synesthesia and went towards a brain function associated with a high level of complexity.

Multi-sensorial perception is mainly modeled as a simple linear summation of inputs or a weighted sum with an almost arbitrary selection of weights. 

The most complex developed model is Hosman’s descriptive model, which has a non-linear combination of visual and vestibular stimuli.

**Figure 4:**
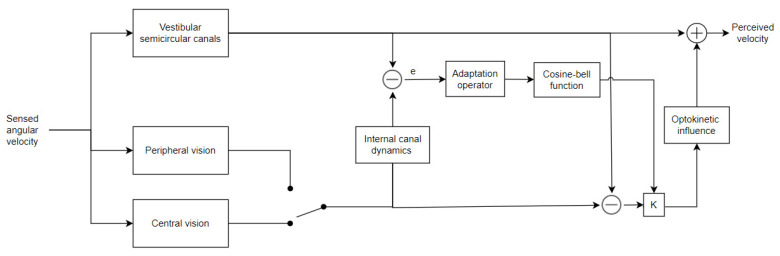
Telban and Cardullo’s rotational perception model.

Telban and Cardullo proposed a model that captured the perception of rotational motion, parametrized to match the latencies experimentally observed in [67,68]. This model can analyze inputs coming from peripheral and central visual fields and vestibular inputs. The rotational perception model, as represented in Figure 4, provides the computation of the perceived angular velocity, given the actual inputs coming from the two considered sensory systems, where the semicircular canals represent the vestibular one. 

A similar model is the translational perception model, in which the perceived velocity and acceleration are obtained based on the actual specific force. In the latter case, vestibular dynamics are represented by the otolith canals, which respond to specific force stimuli. At the same time, the visual system processes the velocity information, which is mathematically represented as an integrated acceleration. In the rotational perception model, peripheral and central vision are considered time delays and set to 90 ms and 150 ms, respectively, in [69]. Further psychophysical experiments have shown that the visual perception of self-movement can induce an artificial vestibular response. The opposite process can also occur, even if to a limited degree.

The ability to represent such an influenced perception of self-movement is the main feature of this model. Optokinetic influence components provide a non-linear gain element and a first-order low-pass filter. The gain element can represent the weights given to vestibular and visual perceptions and is calculated from a cosine-bell function, linking it to the difference between them. The low-pass filter models the semicircular and otoliths canals, implicitly assuming that the CNS compares the visual stimulus with its estimation of vestibular response. 

In vestibular models, a certain degree of correspondence can be noticed between the models proposed by Fernandez et al. [68], Telban et al. [70], and Hosman [69]. In all these models, the otolith organs respond to a specific force, which is defined as:

(7) $$f=\hat{g}-a_{h}$$

Here, $\hat{g}$ represents the local gravitational force vector, while $a_{h}$ is the acceleration of the head of the human operator with respect to a fixed reference frame. Assuming, for the sake of simplicity, that the operator’s head is aligned with the fixed frame axes, it is possible to obtain the transfer function between sensed force $\hat{f}\left(s\right)$ and actual force f (s):

(8) $$\frac{\hat{f}\left(s\right)}{f\;\left(s\right)}=\frac{0.4\left(13.2s+1\right)}{\left(5.33s+1\right)\left(0.66s+1\right)}$$

Meanwhile, in the perceived and actual angular rotations—respectively $\hat{\omega }(s$) and ω (s)—the transfer function can be expressed as:

(9) $$\frac{\hat{\omega }\left(s\right)}{\omega \;\left(s\right)}=\frac{456\;s^{2}}{\left(5.7s+1\right)\left(80s+1\right)}$$

This provides a reliable representation of vestibular canals dynamics. The adaptation operator element indicates the maximum time for which it is possible for there to be a conflict between vestibular and visual inputs by relating the inter-cue error to the washed-out error:

(10) $$\frac{e_{w}\left(s\right)}{\left|e\left(s\right)\right|}=\frac{\tau_{w}s}{\tau_{w}s+1}$$

where $\tau_{w}$ is a time constant. The simulation results [52] showed that the model could represent the difference between the transient nature of the vestibular response and the constant presence of visual stimuli in human motion perception.

## 5. Human–Machine Control Models

This section will discuss human models from a global point of view with a control theory approach. The human–machine system is described in the presented models with a task-dependent approach, typical of control science. Here, significant variations can be noticed regarding the contribution of the physiological structures described in the previous sections as subsystems in the overall model and the level of abstraction of their mathematical representation.

### 5.1. McRuer’s Crossover Model

From the very beginning of the studies in this field, McRuer et al. [71] analyzed human control action in compensatory tasks by randomly changing the target reference trajectory which the human had to follow. The result was one of the most common and simple examples of a human control model, McRuer’s Crossover (CO) model, also known as the quasi-linear model. The quasi-linear model hints at how humans adapt to different plants to elicit stable and effective control responses. 

It can be said that such a model exhibits the behavioral invariance of a human in their adaptation to the controlled machine, offering a consistent human–machine behavior where the functional block diagram can be described to be similar to a simple compensatory manual control system. Due to its simplicity, the proposed model avoids common problems related to higher-complexity systems.

It was observed that when an external disturbance is introduced in the system, the measured human operator responses were different for the different transfer functions of the controlled plant. However, the combined human–machine behavior is approximately the same for all the experiments. The following equation can describe the transfer function of the combined human–machine system:

(11) $$Y_{p}\left(j\omega \right)Y_{c}\left(j\omega \right)=\frac{\omega_{c}}{j\omega }e^{-i\omega \tau }$$

where $\omega_{c}$ is the crossover frequency of the system and *τ* is the overall delay in the human response. Such an equation indicates that the behavior of the human–machine complex can be described as a simple integrator and a delay in the crossover region. If we isolate the human controller from the controlled element, the whole system relating a voluntary motion can be simplified into three components: a linear controller inside the brain, a neuromuscular dynamic, and reaction time delay.

After the learning phase of the machine dynamics is sufficiently finished, the human can be considered a simple feedback controller that moves the controlled element to the target position in the case of a point-to-point task (PTP) by watching the reference target point.

The neuromuscular dynamics, as said before, can often be approximated by a first-order lag, as also demonstrated in [72], and the most straightforward human controller was modeled as a PD controller in [73]; therefore, the human transfer function *Y_p_*(*s*) can be described as follows:

(12) $$Y_{p}\left(s\right)=K_{p}\frac{T_{L}s+1}{T_{I}s+1}e^{-\tau_{e}s}$$

where parameter $K_{p}$ is the pilot’s gain, *T_L_* is the lead time constant, $T_{I}$ is the lag time constant, and $\tau_{e}$ is the pilot’s reaction time delay. The parameter selection is carried out by using the adjustment rules. According to the model, each human subject’s reaction time delay should be constant [74], with small variabilities existing due to task- and environment-related variables. 

**Figure 5:**
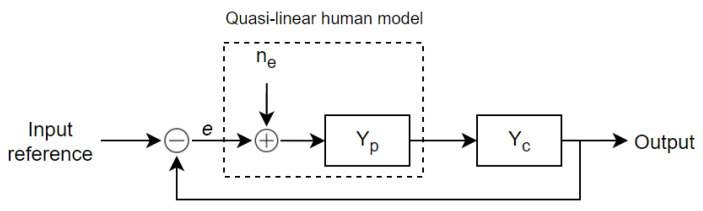
Simple feedback structure for a human–machine complex in manipulative compensatory tasks according to a crossover model.

In the quasi-linear model represented in Figure 5, McRuer introduced the remnant noise term *n_e_* to account for the non-stationary effects of pilot behavior. This remnant was described as a random process that was linearly uncorrelated with the control input. Typically, the remnant is related to the error signal *e*(*t*). It is therefore considered here to be the observation noise.

Despite being created to describe pilot dynamics, the crossover model has become a benchmark in human control models for various applications and controlled elements. For instance, a generalization of the crossover model is proposed in [75], characterizing the human control of systems with both integer- and fractional-order plant dynamics. Alternatively, teleoperated surgical robotic systems [76] can be used for a detailed characterization of operational delay to improve control precision.

### 5.2. Optimal Control Model

The optimal control model was developed by Kleinman et al. [77,78] and Wierenga [79] in the first place due to the advances in optimal control theory made during the 1970s and 1980s. The central concept is that after a certain level of training and motivation, a human operator can control a machine in an optimal manner, even if it remains subject to physical and psychological limitations. The first observable difference from the crossover model is that the optimal control model is expressed using state-space variables, making it easier to extend the human–machine analysis to multi-loop control tasks. 

**Figure 6:**
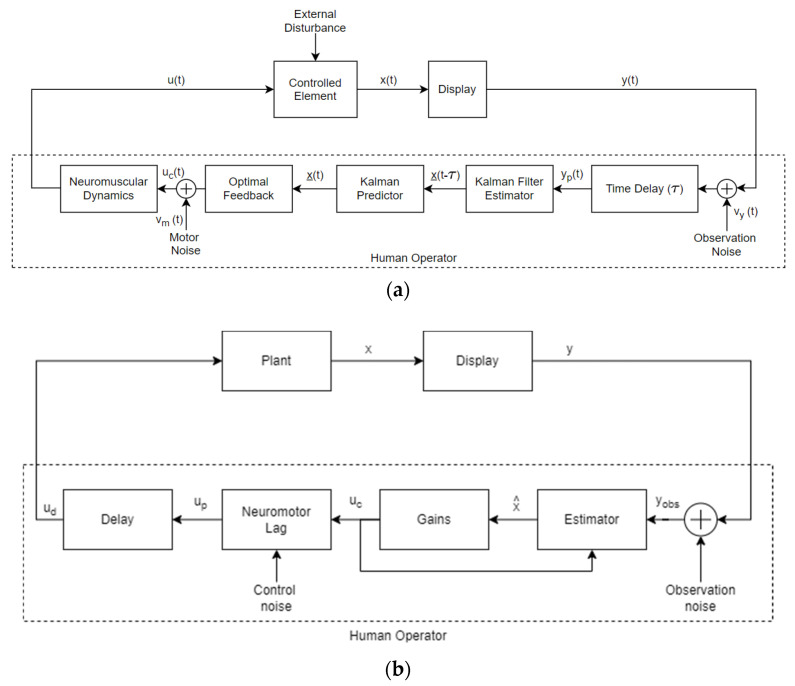
(**a**) Optimal control model of the human operator, as defined by Kleinman et al. (**b**) Modified version of the optimal control model for pilot–vehicle dynamics.

Figure 6a shows the first simple version of OCM. Considering a visual input reference y, the first process is the pilot’s reactive time delay. At the same time, the signal *y_p_* is the perceived input signal—namely, the internal image of the actual input *y* in the CNS of the human pilot. 

Neuromuscular dynamics cause the pilot execute the optimal control and are expressed by first-order lag $\frac{1}{\tau_{N}s+1}$. Moreover, *u* is the output human’s control action, *x* is the internal state vector of the controlled element, *w* is an external disturbance, and *y* is the vector containing externally sensed measurements.

The elements of estimation and decision consist of:

- Kalman filter, which is used to model a human’s ability to deduce a system state from perceived information;
- Kalman predictor, which represents the compensation for inherent time delay;
- Optimal feedback, which builds optimal control *u_c_* based on *y_p_* input.

These elements require a model of the controlled machine and, therefore, can be considered the human’s internal representation of the machine’s dynamics, with all the deriving linearization processes and other psychological limitations [80]. 

The order of the model is dependent on the training level and expertise of the human and can include limited models of actuation systems. The validation of OCM can only be performed with a black-box approach, comparing its output to actual human control outputs. In systems in which a single input is considered, the performance index reflecting human control strategy can be expressed as the following quadratic cost function:

(13) $$J=\underset{T\to \infty }{\lim}\frac{1}{T}E\left\{\frac{1}{2}\int_{0}^{T}\left(y^{T}Qy+g{\dot{u}}^{2}\right)dt\right\}$$

where *E*{} is the expected value, *Q* is the weight coefficients matrix, and g is a real weigh coefficient chosen so that *g* > 0. In manual control compensatory experiments, the element $y^{T}Qy$ is usually set to minimize mean squared error. Here, the determination of *Q* was performed using only empirical methods. Moreover, the factor $g{\dot{u}}^{2}$ sets a superior threshold on the total energy, which can be used in a control task. Including such a term into the cost function results in first-order lag, which is often associated with neuromuscular dynamics [77]. In fact, given the other model parameters, there is a direct proportionality between *g* and $\;\tau_{N}$. Moreover, along with empirical methods, the values of the model parameters were also numerically computed by identifying the OCM model in [81].

However, the model accuracy when matching actual data has not been significantly improved with respect to traditional control models, which indicates a certain over-parameterization. For this reason, OCM has been improved to the form of a modified optimal control model (MOCM) developed by Davidson and Schmidt in [82] and represented in Figure 6b. Another modification developed in parallel led to the fixed-order OCM [83]. Both of them offer transfer function representations with frequency domain analysis. This work can be considered the transition phase between the classical frequency domain and more recent time-domain approaches. However, their complexity is in contrast with the simplification process that motivated their development. Evidence of this concept is that Schmidt himself chose to use the full-parameter model for capturing the effect of aircraft elasticity in his human-in-the-loop simulation and analysis [84].

A revised optimal control model (ROCM) of a pilot based on the aforementioned modified version was also presented in [85]. This model was later extended for the analysis of different aspects of further research works focusing on human decision making in [86], monitoring behavior in [87], the execution of multi-loop tasks in [88], and multimodality in [89], where models of semicircular and otolith canals of the vestibular apparatus are provided.

Overall, the optimal control model was used to solve many applicative control issues, mainly in pilot–aircraft interaction tasks, such as predicting flying qualities [56] and using such predictive capability in refueling tasks [90].

Other research activities were dedicated to the definition of a relationship between Cooper–Harper ratings and the cost function expressed by an equation (shown in Equation (13)) in both single and multi-loop tasks [91,92]. Moreover, the optimal control model has been used for many other applied research activities, such as the simulation of pilot control strategy when encountering a wake vortex [93], the assessment of loads in airframe flights [94,95], or the investigation of display dynamics on the control loop, in order to obtain the relationships between display types [96,97], as well as many other research works [98,99,100,101,102,103].

### 5.3. Structural Model

Despite the successes of the linear models described in the previous sections in investigating the relationship between human control dynamics and handling qualities and their application to analysis/design problems, they both lack an accurate description of the underlying physiological control structure contributing to human pilot dynamics [104]. Moreover, further research conducted in the same period showed that when the difficulty of a control task increases, the human control behavior becomes highly non-linear. 

Hess’s studies were motivated by two main observations: (i) human operator control strategies often seemed to result in discrete or impulsive motions, and (ii) such experimental evidence was not linked to any feature of the classical linear control models. The central assumption of such investigation was that the operator, when associated contemporarily with a high-order-dynamics vehicle and a difficult task, tends to reduce the overall complexity load associated with the time integration of multiple sensory inputs [105]. This simplification is achieved by simply adopting a non-linear strategy relying on a limited number of parameters (rather than a linear strategy associated with a high complexity level and many parameters).

The first development of these assumptions is the isomorphic model [106], which can be considered the father of the successive structural model described by Hess. The main idea is to better describe human signal processing by determining feedback paths from the sensory modalities involved in perception and motor control. The human equalization process, as the human’s “proprioceptive” feedback, occurs through this simulated feedback path, whose parameters are tuned to match the performance of the quasi-linear model near the crossover region [107,108].

**Figure 7:**
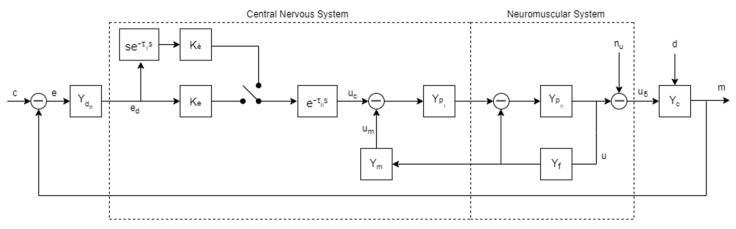
Hess’s structural model of an adaptive human pilot.

Figure 7 shows a schematic representation of the structural model. Here, element $Y_{d_{e}}\;$ is the transfer function of visual dynamics when perceiving its input signal from a display. Moreover, $n_{u}$ represents the remnant noise and, as in the quasi-linear model, is considered an observation noise (and, therefore, put in the human’s output). At the same time, *d* is an external disturbance that acts on the controlled element. The parameters $K_{e}$ and $K_{\dot{e}}$ are the gains of the central processing stage, while τ_0_ and τ_1_ represent the corresponding time delays. In this model, the pulsing logic $Y_{p_{1}}=1$ [109] and element $Y_{m}=\frac{K_{m}}{{\left(s+1/T_{m}\right)}^{k-1}}$ describe the aforementioned pilot inner-loop feedback. A key aspect of this is the selection of parameter k, which can be interpreted as the pilot’s internal model of the controlled element dynamics and reflects the adaptive characteristics of the human pilot. It will mainly depend upon its transfer function around the crossover frequency. 

In the crossover region, it becomes Ym∝s·Yc. Therefore, the following general considerations can be drawn:

- *k* = 0: the controlled element is a constant;
- *k* = 1: the controlled element is an integrator;
- *k* = 2: the controlled element is a square integrator.

The representation of the pilot neuromuscular system includes both front and feedback channels. The describing function $Y_{pn}=\frac{\omega_{n}{}^{2}}{s^{2}+2\zeta_{n}\omega_{n}+\omega_{n}{}^{2}}$ represents the open-loop dynamics of the limb which is driving the manipulator, while $Y_{f}=\frac{K_{f}}{s+1/T_{f}}$ represents the muscle spindles. After its early definition, the structural model was modified, extended, and applied to different scenarios. For instance, the simple application of a structural model in a tracking and regulation task resulted in a motion cue model in [110]. Further experimental activities were directed towards determining time delay effects in manual control systems dynamics. Changes in human equalization performances were observed in the subsequent analytical work because of such time delays [111].

Following several modifications made to the original version of the structural model, Hess developed his revised model in [112], including the effects of the pilot’s neuromuscular system characteristics in the aircraft control process and its ability to perceive forces.

Further extensions of structural models are intended to specify better motion and force feedback [113]. In the aeronautic domain, the Task–Pilot–Vehicle (TPV) model was developed in [114]; this is a simple extension of the structural model that is used for tracking and regulating tasks applied to fast system design. These modifications proved to match the actual data of a human’s describing function in [115]. Such kinds of modified versions of the model were used in many practical control problems, such as the design of a predictive display that also considers motion cues in [116] or the development of an analytical method to assess a pilot’s fidelity in flight simulators [117], including multi-loop tasks [118,119]. The procedure that led to the pilot’s assessment was performed in [120]. A flight was simulated using the six-degrees-of-freedom controller of a rotary-wing aircraft executing a vertical maneuver. A structural model of a human was also used to explore the closed-loop nature of a pilot’s control behavior when determining a target direction and the analysis of the characteristics of pedal feedback to the pilot [121]. Finally, evaluations of aircraft handling qualities were proposed in [122,123]. Their prediction, along with the estimation of pilot-induced oscillations, was applied to different controlled elements in the following years [124,125,126,127].

### 5.4. Descriptive Model

In Europe during the 1970s, technological developments such as the Fly-By-Wire (FBW) aircraft aroused new research interest in man–machine interaction modelling for aeronautic scientists. Researchers started to consider their human modelling efforts in order to better understand flying handling qualities and performances [128,129] and improve the accuracy of flight simulation. For this purpose, the better integration of visual and vestibular systems into the control model seemed necessary.In the 1990s, Hosman led extensive research efforts aiming to understand the influence of the visual and vestibular systems on human perception and, consequently, control behavior [130]. This investigation resulted in the definition of the descriptive model. To do so, Hosman presented different experimental works in which the case study used was the pilot’s landing maneuver with an aircraft using a moving-base flight simulator [131]. In this, systematic variations in sensory inputs were the base that led to the definition of the descriptive model [132]. Results were applied in closed-loop control tasks where the human was considered a single-channel information processor with multiple inputs from the sensory systems.

**Figure 8:**
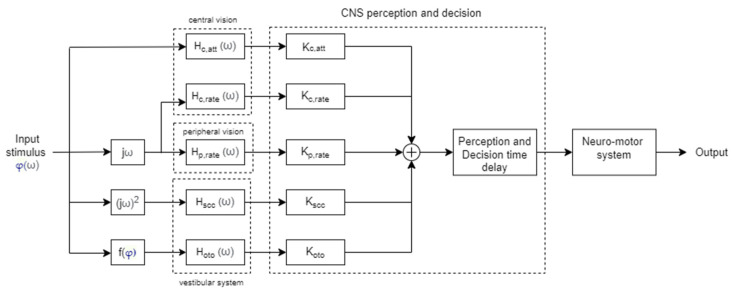
Hosman’s descriptive model of human control behavior (1996).

The descriptive model represented in Figure 8 has a multimodality structure, reflecting physiological subsystems that link the state of the controlled element to their perceived state. Here, the visual perception of displacement is expressed by the time delay $\;H_{aat}\left(s\right)=e^{-\tau_{att}s}$, where the attitude perception delay $\tau_{att}$ was found to be around 50 ms. The visual perception of velocity is $H_{rate}\left(s\right)=e^{-\tau_{rate}s}$, where the delay parameter varies in the case of central or peripheral vision. In particular, the peripheral system can only sense rates; therefore, its dynamic is described by the second equation, but with a shorter time delay (60 ms) with respect to the one measured in the central vision system (110 ms) [133]. Similarly to the time delay referred to by McRuer in his crossover model is the sum of the delay associated with the detection of the stimulus in the eye and the one associated with information processing.

Regarding the vestibular system, the semicircular and otholit canals are modeled, respectively, as an over-damped torsion pendulum and an accelerometer with over-damped mass-spring-damper characteristics. They can both be represented by second-order differential equations with the following transfer functions: $H_{scc}\left(s\right)=\frac{\left(1+\tau_{L}s\right)}{\left(1+\tau_{1}s\right)\left(1+\tau_{2}s\right)}$ and $H_{oto}\left(s\right)=\frac{\left(1+\tau_{n}s\right)}{\left(1+\tau_{a}s\right)\left(1+\tau_{b}s\right)}$. In his model, Hosman assumed that tactile and proprioceptive senses were implicitly considered within the vestibular dynamics model.

Moreover, the descriptive model assumes that processing in the perception and decision stages by the central nervous system and neuromuscular dynamics can be combined and represented by a unique “information processing” transfer function:$\;H_{ip}\left(s\right)=K_{ip}e^{-\tau_{ip}s}$. Here, the CNS contributes to both the gain and delay elements, while only a delay factor approximates the neuromuscular dynamics low-frequency effects; thus, *τ_ip_* is the sum of the delay contributions of both factors. Function f converts the displacement caused by the input stimulus to a specific force output.

The descriptive model has been applied in numerous studies in the transport engineering field focused on the identification of a human pilot’s dynamics, such as the implementation of optimal forcing functions for identifying human model parameters [134], the investigation of the use of visual information by the operator while controlling an aircraft [135], or the study of the influence of translational movements on a pilot’s performance and perception [136,137].

### 5.5. Biodynamic Models

Biodynamic models were developed to represent the effects of the body dynamics on the human’s desired control input in a situation in which they are subject to an accelerating environment. This way, the effects of this kind of motion on human health, comfort, and performance can be predicted [138]. Human control actions can be divided into two general categories: voluntary and involuntary [139]. The models studied in the previous sections all belonged to the first category. All of them focused on translating the process which converts an idealized voluntary human action into an actual control action into mathematical expressions. This process considers aspects such as bandwidth limitations and delays in human dynamics in relation to rigid-body movements and human-induced oscillations.

An alternative modeling technique aims to describe involuntary human actions as a direct consequences of the vibrations coming from the environment, which are filtered by the human’s body and become an involuntary input into its control system [140,141,142,143]. 

The modelling of biomechanics can be categorized into three types: continuum, discrete, and lumped parameter models. The main difference between them is how the spine is modeled.

**Figure 9:**
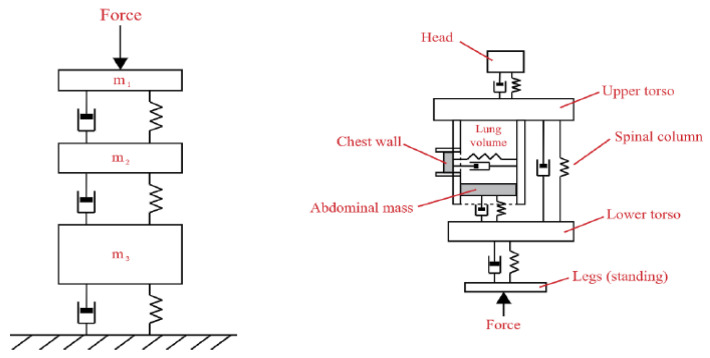
Examples of discrete (**left**) and lumped parameter (**right**) model structures.

In continuum models, the spine is considered a flexible beam; its responses to vertical accelerations were studied by Griffin et al. [138]. Meanwhile. the spine is described as a series of interconnected mass-spring-damper elements in discrete models. The dynamic response can be studied by determining its equation of motion. With respect to the continuum modelling approach, discrete models represented in Figure 9a, succeed in describing the human body as a composite of elements (i.e., organs), each one with its own resonance frequency. 

The body itself is modeled as an equivalent mass-spring-damper system in the lumped parameter model, depicted in Figure 9b. Of course, this approach causes models of this type to have only one or two degrees of freedom. Moreover, the efficacy of its analysis is limited to the response to vertical stimuli [144]. It is not able to capture the complex dynamics of the human body, as evidenced by Sirouspour et al. [145] when attempting to model the lateral dynamics of a seated subject. This kind of approach can avoid instabilities and cancel dynamic feedthrough [146]. A further aspect that must be considered in biodynamic modelling is whole-body transmissibility [147,148], which is the ratio between the vibration measured at a particular point of interest and the base vibration (both are a function of frequency).

The area which has produced the most research activities in this field is, once again, the aerospace domain. In [149], a simulated biodynamic model was implemented to predict both the human dynamic response and tracking performance in vibrating environments, allowing the researchers to gather data on whole-body vibration. Based on this model, further experiments were conducted to simulate the transmissibility of vertical base vibrations to lateral and roll accelerations [150,151]. One of the first important studies focusing on biomechanical effects associated with human–manipulator interaction at high frequencies was carried out by Johnston et al. in 1988 [152]. 

This century’s modern technological developments, which are characterized by the higher speed of transport systems, have motivated further research into the effect of the structural vibrations of civil transport [153] and supersonic aircraft [154]. Later, muscular damping and stiffness parameters were studied with regard to the urgency of the performed task [155], suggesting that during tasks that are perceived to have a higher urgency level, the body stiffness increases (this can affect such factors as a pilot’s grip force). Moreover, in [156], a three-dimensional human body model which relied on collected experimental data was defined. Meanwhile, in [157] the error between the human’s intended and actual control action was investigated and put in relation with the biomechanical models of the limbs.

## 6. Human–Machine Interfaces

The study of human–machine interaction has developed, along with modern technological advances, into the study of interfaces able to maximize the exchange of information between the operator and the controlled element, and, therefore, a human’s capability to understand system dynamics. This is currently one of the most active research fields dealing with human control behavior modelling. One of the application scenarios which has led to such research activities is autonomous vehicles. 

Eskandari et al. [158] studied human perception characteristics and limitations when interacting with a machine, describing them in terms of the observability and predictability of the human–machine control system. In doing so, the user was modeled as a function able to map the information obtained from the controlled part (through a display) into its control input. Human information processing stage has the role of providing sufficient knowledge about system dynamics to the user. 

**Figure 10:**
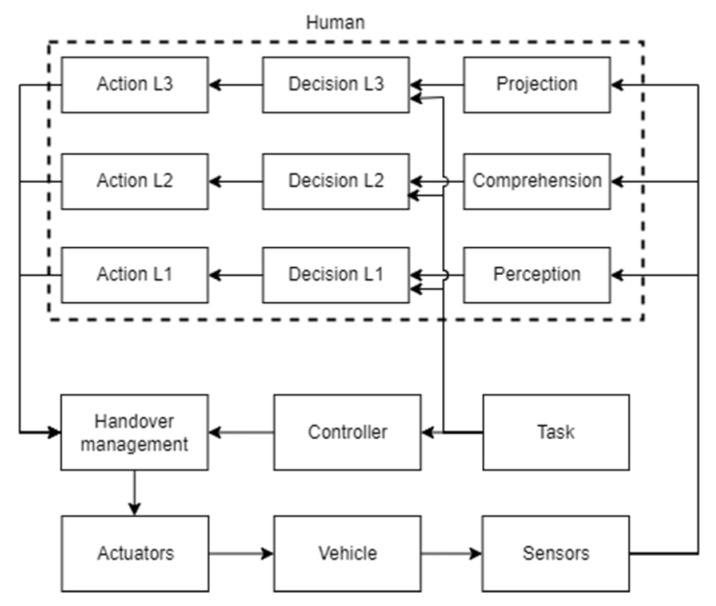
Driver control model for guiding an autonomous vehicle studied by Drexler et al.

Drexler et al. [159] proposed a model able to unify the Eksandari approach with the classical optimal control model for measuring human Situation Awareness (SA) when interacting with an autonomous vehicle. As shown in Figure 10, the driver’s behavior is described using three different SA levels: perception, comprehension, and projection. The level which is used for a specific task depends on the available processing time. Each SA level is modeled as a time delay $\tau_{SA}$ with the transfer function $e^{-i\omega \tau_{SA}}$, which increases proportionally to the level itself. The decided control action is then carried out through the neuromuscular dynamics, described as a second-order transfer function plus a time delay element.

The most interesting step towards the development of advanced interfaces between human and machines is related to haptic feedback. Haptics can be described as an extension of standard bidirectional devices such as displays, which provide only visual information. On the other hand, the objective of haptic interfaces is to increase the human sensation of their presence in the external environment through force, inertial, and vibration feedback information. 

Teleoperated systems are a classical application scenario in which this kind of approach has proved to be more efficient. In bilateral teleoperation, for instance, the physical coupling between a human and the environment is very strong and based on the exchange of low-level continuous time signals. Several research activities have focused on the improvement in the system stability and transparency of these kind of systems, particularly in the robotic field [160,161]. 

The characterization of human dynamics is a crucial aspect of the design of these kinds of systems and is able to affect their stability and performance. Takács et al. [162], for example, formulated a model of tool–tissue interaction, aiming to improve the performance of a telesurgical robotic system with haptic force feedback.

Hirche et al. [163] proposed a generalized scattering transformation technique able to consider the damping properties of a human arm and manipulator in the design of a passivity-based controller with the presence of time delay.

## 7. Conclusions

The study of human control behavior models when interacting with a controlled machine has played a crucial role in many fields of engineering. Emerging technical challenges, such as the development of new robots, aircraft, and vehicles, or the spread of advanced simulation frameworks, have motivated the rise of more and more complex mathematical representations over the years. Along with the rise of the complexity of applications, the level of abstraction of human behavior in terms of skill has also increased, making the objective of including the accurate functional modelling of the involved physiological structures more difficult. For this reason, the parallel research efforts performed in motor control theory and sensory feedback applications were reviewed in this work.

The open discussions had in cognitive-science-related research activities were also detailed in this paper, regarding the presence of the internal models of external dynamics with which a human is interacting in their central nervous system. Corresponding differences in the functional description of the motor apparatus were also identified. Regarding this review of human sensory modelling, we included subjects such as visual perception errors, their modelling, and vestibular and proprioceptive sensory dynamics.

Neuromuscular dynamics representations completed our discussion on the control theory representation of involved physiological districts, considering the modelling efforts of internal feedback loops provided by tendons and spindles, forward muscular activation dynamics, and their approximation in the frequency range of a manipulative control task.

Passing to a task-based description of the human–machine complex, the aforementioned physiological dynamics were included in more general control structures. Different higher-level human features were also represented. Thus, humans’ ability to behave optimally after a certain level of training was represented in the optimal control model, while its adaptability to the machine dynamics was captured well by the classical crossover model or by Hess’ structural model.

The degree of integration of the underlying physiological processes and mission-based strategies used within these models was variable, with the advantage of the structural model and Hosman’s descriptive model being discussed with respect to the previous classical quasi-linear approach. Ever-more-complex applications are motivating the development of the higher-level representation of human control strategies and decision-making, including techniques taken from fields such as robust control theory, uncertainty propagation, and probabilistic methods. Therefore, maintaining a bond between these two needs will become increasingly challenging.

In accordance with the relevant research efforts made in the last few years to describe the non-linear dynamics involved in man–machine interaction, the reader is encouraged to face this topic starting from the simple models overviewed in this paper that have the advantages of being able to be handled using the classical approach of linear system theory and automatic control methods.

## Figures and Tables

**Table 1:** Examples of existing state-of-the-art classifications of human behavior described by control modelling techniques.

Rasmussen Behavior Classification	Xu et al. Models Classification	Control Models
Skill-based	Based on control theory Based on human physiology	Crossover model Optimal control model Structural model Descriptive model Biodynamic models
Rule-based Knowledge-based	Based on intelligent techniques	Fuzzy control models Neural network models Models based on other machine learning techniques
